# The evolution of the human healthcare system and implications for understanding our responses to COVID-19

**DOI:** 10.1093/emph/eoac004

**Published:** 2022-02-12

**Authors:** Sharon E Kessler, Robert Aunger

**Affiliations:** 1 Department of Psychology, Faculty of Natural Sciences, University of Stirling, Stirling, UK; 2 Environmental Health Group, London School of Hygiene and Tropical Medicine, London, UK

**Keywords:** evolutionary medicine, animal behaviour, primatology, human evolution

## Abstract

The COVID-19 pandemic has revealed an urgent need for a comprehensive, multidisciplinary understanding of how healthcare systems respond successfully to infectious pathogens—and how they fail. This study contributes a novel perspective that focuses on the selective pressures that shape healthcare systems over evolutionary time. We use a comparative approach to trace the evolution of care-giving and disease control behaviours across species and then map their integration into the contemporary human healthcare system. Self-care and pro-health environmental modification are ubiquitous across animals, while derived behaviours like care for kin, for strangers, and group-level organizational responses have evolved via different selection pressures. We then apply this framework to our behavioural responses to COVID-19 and demonstrate that three types of conflicts are occurring: (1) conflicting selection pressures on individuals, (2) evolutionary mismatches between the context in which our healthcare behaviours evolved and our globalized world of today and (3) evolutionary displacements in which older forms of care are currently dispensed through more derived forms. We discuss the significance of understanding how healthcare systems evolve and change for thinking about the role of healthcare systems in society during and after the time of COVID-19—and for us as a species as we continue to face selection from infectious diseases.

## Lay Summary

The COVID-19 pandemic revealed a need to understand how healthcare systems respond successfully to pathogens – and how they fail. To understand this problem, we develop a novel evolutionary approach which can be applied across species. We trace the evolution of care-giving and disease control behaviours across species, mapping out how they likely integrated in our lineage to form the foundation of the healthcare systems we have today. We then use our evolutionary perspective to analyse our behavioural responses to COVID-19 and find that three types of evolutionary conflicts are occurring that make our healthcare systems vulnerable to failure: 1) conflicting selection pressures on individuals, 2) mismatches between the context in which our healthcare behaviours evolved and our globalised world of today and 3) displacements in which evolutionarily older forms of care are dispensed through more recently evolved forms. This paper presents the first theoretical framework for tracking the evolution of healthcare systems across species. It has implications for understanding our responses to COVID-19 and for thinking about the role of healthcare going forward.

## INTRODUCTION

In 2020 COVID-19 swept across the globe, overwhelming healthcare systems and forcing many nations to shut down economies to reduce transmission. Now, roughly 20 months later, the world is convulsed with repeated waves of outbreaks and consumed by debates on how to distribute supplies for treatment and prevention (e.g. [1–4]). In addition to the urgent need to slow the waves of outbreaks and reconfigure healthcare systems to be more resilient to future pandemics, the requests for behavioural changes (i.e. lockdowns, social distancing and mask wearing) have forced populations to think about global disease transmission dynamics to a degree they did not before (e.g. [5–8]). This focus on global health—by more than just health experts and policy makers—means that how populations understand their experiences of the pandemic and incorporate this into their worldview has the potential to shape public support for healthcare initiatives for years to come.

Although building consensus has been difficult, partly due to scientific, political, and cultural conflicts, the scientific community is working towards a multidisciplinary understanding of how and why the COVID-19 pandemic unfolded as it did. Biomedical (e.g. [9–14]), public health (e.g. [15–19]), health psychology (e.g. [20, 21]) and cross-cultural [22–24] researchers are analysing our responses to the pandemic and producing recommendations for how to reconfigure our health systems to better withstand pandemics. The public is participating in these discussions through their individual and collective responses to public health policies, i.e. compliance with lockdowns and contact tracing, vaccine uptake or resistance, pandemic-related protests, voting, and so on (e.g. [5, 7, 8, 25]).

Our study contributes to these discussions by illuminating the evolutionary context in which our healthcare systems evolved. The goal is not to produce recommendations for controlling the COVID-19 outbreak, but to understand the selective pressures that shape healthcare systems over evolutionary time. This is relevant because disease outbreaks have exerted strong selective pressures on our healthcare systems—and are continuing to do so today as we respond to COVID-19.

We trace the evolution of behavioural strategies for controlling disease across species—that is, the evolution of what we call the ‘healthcare system.’ We borrow from the primate behaviour literature to develop a new, definition of ‘the healthcare system’ which is informed by the theoretical approaches of socioecology and evolutionary biology (i.e. [26, 27]) and applicable across species. This allows us to track the evolution of behaviour patterns across species, revealing a surprising amount of continuity through evolutionary time. This theoretical advance facilitates novel analyses for how different behavioural strategies may have been shaped by natural selection and how they may interact producing a ‘system.’ Similarly to how social systems are understood to be emergent effects of individual behavioural interactions [26–28], healthcare systems can be understood as the emergent effects of individual interactions with conspecifics, with pathogens, and with the environment in health-relevant contexts. It is also important to note, that because healthcare systems are emergent properties of the behaviours of individuals, they can result from the selection that occurs largely at the level of the individual. In other words, healthcare systems can result from selection without, themselves, needing to die or reproduce like biological entities.

Our definition of a healthcare system does not prioritize (or exclude) the highly technological, biomedical healthcare system that is currently dominant in human societies. Instead, it situates the healthcare system as one, albeit highly complex, system with unusual traits that require explanation. In doing so, our study maps which elements of human healthcare systems are unique to us and how they have been a key part of our success as a species. We then place our behavioural methods for controlling COVID-19 into this evolutionary framework, examining how the evolutionary processes driving the evolution of healthcare systems creates conflicts within these systems. We highlight the evolutionary pressures that make the modern healthcare system vulnerable to breaking down—including during our response to COVID-19. We discuss the significance of understanding how healthcare systems evolve for thinking about the role of healthcare systems in society, during and after the time of COVID-19.

This study will therefore:

1. Track how the behavioural strategies for disease control observed across the animal kingdom evolved into the components of human healthcare systems.
2. Situate our responses to COVID-19 within this evolutionary framework.
3. Map the complex selective pressures that create conflicts between the components of our healthcare system, including during our responses to COVID-19.
4. Open questions about how COVID-19 may cause us to debate and reconceptualise the role of healthcare, not only in society today, but also for us as a species over evolutionary time.

## EVOLUTION OF THE HUMAN HEALTHCARE SYSTEM

Humans, like all living things, have co-evolved with pathogens. Selection pressures to combat diseases are ubiquitous, stimulating species to evolve complex batteries of defences [29]. A comparative, cross-species approach allows us to track how and when these defences evolved and how they fit together today—in both nonhuman and human animals.

Defences against infectious diseases are often divided into the physiological and behavioural immune systems, with the physiological immune system serving primarily to defend the body against infections after exposure [29–32]. Its complement, the behavioural immune system, evolved to prevent exposures to disease and to supplement the physiological immune system when infected [30]. However, the behavioural immune system concept is limited to individual-level psychological and behavioural responses to cues of disease (i.e. disgust responses [30, 33–35]). This study will also trace the evolution of cooperative group-level defences which have evolved convergently in eusocial insects and humans [36–38]. The analysis will highlight both the similarities and the differences between species’ defence systems, including how cooperative defences may fail in humans because of the ways we are different from eusocial insects.

**Figure 1.**
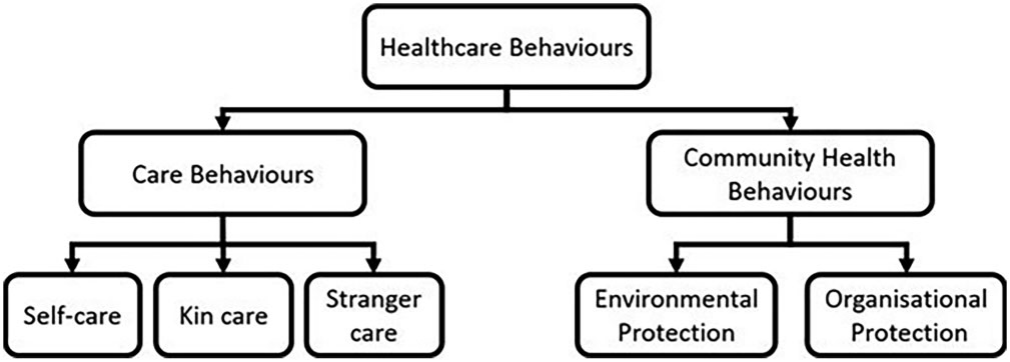
A conceptual diagram showing the hierarchical relationships between the terms used in the study. This diagram does not show evolutionary pathways, just the relationships between the terms

Here, we refer to behavioural defences as healthcare behaviours [39] and divide them into two overarching categories based on how they operate: care behaviours and community health behaviours [39]. Care behaviours refer to behaviours that benefit the health of a targeted individual (who is often sick). We subdivide care behaviours into self-care, kin care, and stranger care based on the relationship between the carer and the recipient. These behaviours do not require compassion or empathy. Community health behaviours generate indirect benefits for the group through actions which are not directly targeted at a sick individual. We subdivide community health behaviours into environmentally-mediated protection (environmental protection) and organisationally-mediated protection (organizational protection). Environmental protection consists of actions that make the environment more hygienic and hence less favourable to the growth of pathogens. Organizational protection includes subgrouping of behaviour patterns in space or time in ways that reduce opportunities for transmission, e.g. divisions of labour, synchronization of hygiene behaviours, and so on. The different types of healthcare behaviours which benefit others (kin care, stranger care, environmental protection, and organizational protection, discussed below) are categories of behaviours which can produce group-level defences like social and organizational immunity [36–38]. These different categories of behaviours are useful because they highlight how they may be driven by different selective pressures and/or occur in some species but not others. The distinction between care and protection also closely mirrors the common medical contrast between treatment and prevention. Figure 1 is a conceptual diagram showing the hierarchical structure of these definitions.

We also focus on socially transmitted infectious diseases, although our model for the evolution of the healthcare system does not exclude responses to non-infectious diseases or injuries. Individuals are likely to be under selection to distinguish between infectious and non-infectious conditions, but are unlikely to do so with perfect accuracy [39]. Therefore, the evolution of care is likely to include responses toward individuals suffering from both infectious and non-infectious conditions, as the aetiology of a condition is not always distinguishable [39]. Because non-infectious conditions are less costly to carers (as they won’t be infected), the inclusion of care for some non-transmissible conditions should reduce overall selection against care [39], making care more likely to evolve.

**Figure 2.**
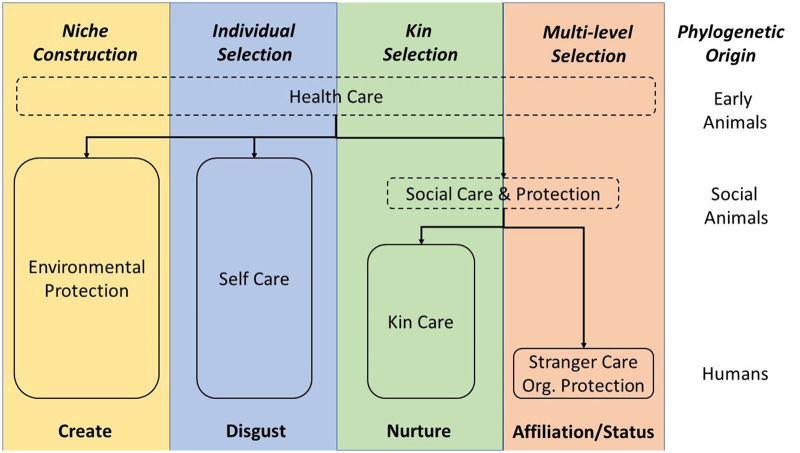
A conceptual diagram showing the elements of the human healthcare system and how they evolved. The different selective processes are colour coded and named across the top. These processes have produced the elements of the healthcare system shown below in boxes. The phylogenetic origins of the different types of care are shown on the right. Early animals, social animals and humans refer to the taxa in which certain behaviours are hypothesised to have evolved, although once evolved, each type of behaviour has persisted into the present day. Niche construction is a special kind of feedback into the selective regimes derived from factors in modified environments. We depict multi-level selection as the primary selective process for stranger care and organisational protection, but this does not exclude contributions from forms of reciprocity. The psychological motivations producing the corresponding behaviours are shown across the bottom [40]

Figure 2 is a conceptual diagram which summarizes the different kinds of care present in humans, their phylogenetic origins, the underlying selection processes, and their psychological motivations. Most of the behavioural defences have deep evolutionary origins, although stranger care is uniquely human. Table 1 highlights the striking parallels that exist between our defences against diseases (such as COVID-19) and their counterparts in other taxa. We next discuss this evolutionary history in greater detail.

**Table 1.** Examples of convergent evolution between healthcare behaviours in humans and eusocial insects, birds, and mammals

Care type	Humans	Eusocial insects	Birds	Mammals
Self-care	Avoiding others who are infected, handwashing	Secreting antimicrobial/antifungal substances, avoiding infected individuals	Self-grooming; avoiding infected individuals	Medicinal plant use, self-grooming, avoiding infected individuals
Environmental protection	Latrine use, disinfecting public areas	Using antimicrobial/antifungal secretions in nest construction, nest sanitation	Nest hygiene, i.e. removal of egg sacs, building nests with anti-parasititic/anti-microbial materials	Latrine behaviours, building nests with antiparasitic materials
Kin care	Providing food, water, shelter, hygiene assistance and basic medical care	Providing food, water, shelter, and hygiene assistance and medical care: social grooming, including coating nestmates with antimicrobial/antifungals, transporting wounded nest-mates back to nest and cleaning their wounds to reduce infections	Grooming kin	Providing food and shelter to individuals who cannot forage, providing protection (standing over those who cannot move) or lifting them out of water to breathe
Stranger care	Individual care specialists (e.g. healers, midwives)	N/A	N/A	N/A
Organisational protection	Division of labour between carers and noncarers; spatio-temporal segregation between infected and unexposed (i.e. isolating vulnerable groups; synchronized group-level responses like group-level lockdown, closed national borders, contact tracing)	Division of labour among those engaging in at-risk activities; spatial segregation of those engaging in at-risk activities by area or life stage; refusing entry to infected nestmates and outsiders; isolating individuals by age category (larvae) and status (queen); abandoning an unhygienic nest; subgroups who specialize in disposing of corpses and waste	N/A	N/A
Citations	[16, 17, 41–53]	[36–38, 54–56]	[57–64]	[39, 65–73]

## CARE BEHAVIOURS

First, we discuss the evolution of the three types of care behaviours: self-care, kin care, and stranger care. While the evolutionary history and selection pressures driving of each type of care differ, many of the specific behaviours may be directed to both the self and others (e.g. grooming). This highlights that both the behaviours and the decisions of whom to direct the behaviours to, were likely targets of selection.

### Self-care

The oldest form of care is self-care. Individuals have had an evolutionary incentive to take care of themselves—to repair damage to their bodies and avoid infection, where possible—since the beginning of life on Earth. Thus, self-care is the foundation of any care system. Notably, many self-care behaviours, i.e. grooming, are also given to others as forms of social care in social species. Widespread self-care strategies include self-grooming (insects [54], birds [57], mammals [74]) and pathogen avoidance behaviours (i.e. lobsters [75], birds [58], insects [55], and primates [76, 77], reviews [65, 78]). These self-care behaviours are deployed widely by social and non-social species alike under many different environmental conditions and in response to many different pathogens. Accordingly, the proximate mechanisms by which infections are recognized in others are highly variable, including detection of chemical [79, 80], visual [80, 81], auditory (i.e. coughing, sneezing), and behavioural [29, 31, 82] cues depending on the pathogen involved, the habitat, and the sensory abilities of the species. Importantly, by triggering generalized fear, disgust, and neophobia responses, the organism does not need to have a concept of what a disease is in order to effectively avoid it [33, 34, 40, 78, 83]. In sum, this broad range of host taxa, multiple proximate mechanisms allowing recognition of diverse infections (or their indicators), and basal, ubiquitous psychological processes underpinning them (citations above), suggest that (1) self-care is likely to be the most ancestral component of healthcare systems and (2) these basal strategies of self-care have been maintained across lineages as they evolved and diversified into different ecological niches.

### Kin care

Once kin-based sociality evolved (for whatever reason) and related individuals grouped together, it became possible to provide care to kin. Kin care behaviours benefit the genetic relatives of the actor by helping infected kin recover [84–88]. Presumably, specialised motivations for nurturing dependent relatives evolved to support such behaviours [40]. Multiple authors have suggested that care-giving for the sick evolved as a generalisation and co-optation of the care given in infant rearing systems [89–91]. Among mammals and social birds, there is frequently a high degree of overlap in infant care networks and kin care networks, while in eusocial insect societies brood care and care for infected/injured foragers may be done by different individuals, in different locations in the nest, as part of the division of labour which protects the brood from transmission [38]. Due to intense selection on individuals to care for young, these proximate mechanisms for facilitating offspring care are easy to activate and co-opt, making them likely to also be the foundations of care-giving and altruism more broadly, including care for sick adult relatives [89, 91]. There is also evidence showing that (i) much of the care that is given (even among humans) is given along kin networks [92], and (ii) behaviours given to sick and vulnerable kin (social grooming, provisioning, guarding/carrying) are typically also given to healthy, but defenceless offspring [39, 89].

Kin care behaviours have received most research effort in three major taxonomic groups: eusocial insects, mammals, and birds [39]. Across these groups we see extensive overlap in the types of care given to young and to sick/disabled individuals [39]. Examples include eusocial insects allo-annointing immatures (larvae) and mature nest-mates with antimicrobial/antifungal secretions [54]; cooperatively breeding mammals provisioning sick/injured/disabled individuals, similarly to their treatment of young [66, 93]; and nestlings who allofeed and allopreen their siblings even as they sit in nests waiting for similar types of care from their parents [59]. While these examples are striking, the strongest evidence comes from comparative, phylogenetically controlled studies which allow researchers to test for evolutionary associations between kin care behaviours and infant rearing systems. For example, Kenny and colleagues [94] showed that allopreening is associated with parents cooperating to raise young.

The origins of kin care in the human lineage are difficult to pinpoint, but alloparenting (carrying infants, providing food and protection from predation by individuals other than the parents) is common among mammals, especially primates [95]. Monkeys may accept unknown, injured individuals into their group [96, 97] and apes engage in low levels of kin care [i.e. guarding the sick, dying, or dead [73, 98–101]]. It is therefore likely that ancestral hominins engaged in at least as much kin care as other primate species do today. Ancestral hominins are argued to have shifted to a strategy of cooperative breeding [102–104] with increased birth rates leading to greater numbers of dependent young [105] with immature immune systems. Based on the links between infant rearing systems and care for sick kin in other taxa, a shift to a more intensive breeding system may have produced a corresponding increase in care for sick kin (who were necessary for cooperative infant care).

This shift to cooperative breeding may have also been associated with a shift to ‘obligate midwifery’ [106]. Despite group members frequently being nearby during births, assistance during birth is rare in nonhuman primates though it is nearly universal in human populations [107]. The frequency of birth assistance in human populations combined with difficult (though not uniquely human) birth mechanics, has lead researchers to propose that birth assistance was an early evolving form of care which was important for reducing infant and maternal mortality during birth [107]. This care was likely to have been given by kin (i.e. older females), mates, or other trusted individuals [106].

Fossil evidence of care is generally lacking, however. The fossil record includes hominins who survived debilitating conditions [108–111], but while it is possible that they received care, comparative studies showing that wild primates have survived similarly debilitating conditions without care [112–118], meaning that we cannot be certain that care was given to these individuals.

Care for kin has evolved in both individualised societies in which individuals are able to recognize other individuals (i.e. mammals, social birds) and in eusocial insect species with anonymous societies [119] which may not even discriminate between different genetic lineages of nest-mates [120]. Species that form long-term individualised social relationships may be more likely to use brain pathways linked with social cognition to detect the appearance of unusual symptoms, i.e. coughing or changes in vocalisations during a respiratory infection, behavioural changes like increased lethargy, odour changes, or changes in colouration due to fevers or rashes, etc. [121–123]. (Humans have even been proposed to have an emotion which coordinates defences against infections, including signalling a need for care from trusted individuals [123], see also Signalling Theory of Symptoms [124–126]). On the other hand, eusocial insects detect infection/injury/death based on multi-modal cues [54–56], such that the underpinning cognitive processes are not likely to require a prior relationship with the infected individual.

### Stranger care

The next extension of the ‘expanding circle’ of care is to unrelated group members. Humans are the only species which have a specialised system for providing care specifically to unrelated strangers. Many species live in large groups, but do not have a specialised care system to care for unfamiliar, unrelated individuals. Across the animal kingdom, there are examples of care being given to unrelated strangers (even of another species): birds exploited by brood parasites [127], insect colonies that are parasitised by social parasite species (i.e. slave-making species [128]), and eusocial insects with multiple genetic lineages in a nest that do not discriminate by kinship [120]. However, these do not count as examples of adaptive stranger care because each of these cases is likely due to a discrimination failure (and/or reciprocity, mutualism, etc.), not a distinct system of care for strangers. Here, we place stranger care in humans within an adaptive framework, however, we do acknowledge that stranger care could have also evolved as a by-product [129] of kin care which then intensified to become an adaptive component of the human healthcare system.

How stranger care evolved, and why it evolved only in humans, is puzzling. Due to inclusive fitness [88] in the form of shared genes, this kind of care depends on reciprocation of care or some other form of remuneration to persist, as the practice is costly to the carer, energetically and in terms risking their own health, and by extension, the health of the close kin with whom they will be in subsequent contact. Disease transmission models show that while kin care has the advantage of improving recoveries while limiting transmission to those outside the close kin-group, stranger care increases cross-kin group transmission [85]. It would therefore appear that group-level selection would also work against stranger care unless there were other group-level benefits, such as group-level reductions in infectious transmission rates, possibly thanks to more skilled and effective care provided by experts. These models also suggest that kin care may be a prerequisite for the evolution of stranger care; kin care may be necessary to deal with the increased transmission caused by cross-kin group infections that can arise from stranger care [85]. In other words, if the stranger carers (i.e. healers) get infected while giving care, they, themselves, will need care from others, including kin, while they recover. In this way, kin care supports the evolution of stranger care by helping infected stranger carers.

The models also indicate that it is easier for stranger care to become established in smaller, less dense groups [85]. This evidence, combined with our knowledge of stranger care in modern forager-horticultural communities ([130, 131], and see [132] for an example of medicinal plant knowledge primarily, but not exclusively shared along kinship lines) suggests that stranger care networks can exist without biomedical institutions in low-density, small-size groups. Evidence from modern small-scale societies also suggests that small-scale stranger care can be motivated and maintained by economic reciprocity [130, 131] and cultural transmission [132], opening the possibility that it may have been similar in ancestral small-scale populations.

The proximate motivations and evolutionary mechanisms underlying stranger care are not yet fully understood. A primary, proximate motivation may have been reputational benefits, particularly if being a successful carer yielded status or other fitness enhancing benefits (power, prestige, wealth, access to mates, etc., for data from cross-cultural work [133]). Potential links between healing and religion may have also reinforced these dynamics [134, 135]. Interestingly, cross-cultural analyses of ethnoscientific expertise suggest that medical knowledge for dealing with uncommon, but serious events are associated with secretive and proprietary behaviour [133]. This contrasts with the teaching behaviours that were associated with sharing subsistence and technical skills [133]. We hypothesise that stranger carers may have offered specialised skills for dealing with uncommon conditions which kin carers may have been unable to treat.

The evolutionary mechanisms involved were likely complex, not mutually exclusive, and may have changed over evolutionary time depending on the social dynamics and epidemiological conditions. It is also conceivable that at times, stranger care may have been maintained by cultural dynamics, even when there was a net cost to the carer. Possible mechanisms creating selection for stranger care include direct reciprocity (carer receives a benefit in exchange for care), indirect reciprocity (carer receives a benefit, though not from the individual who received care), network reciprocity (a cluster of individuals provide care to each other), or multi-level selection (group-level benefits make groups with care able to outcompete groups that with no carers) [136]. We expect that the combinations of mechanisms which supported the evolution of care were likely highly dynamic—due to continually changing epidemiological and social conditions.

This complex and potentially continually changing balance of selective pressures may be the ultimate reason why stranger care is especially vulnerable to failures of cooperation. Unlike kin care, where there are clear, inclusive fitness benefits to providing care, the benefits of providing stranger care can be indirect, dependent on the cooperation of many others, and delayed in time until cumulative, emergent effects result (i.e. group-level benefits). This makes stranger care especially fragile and may be the reason why it has not evolved in more species and even in our species, often gets reinforced by immediate direct benefits to the carer (i.e. payment from the recipient).

However, through these processes, stranger care does appear likely to have evolved as an element of increasing economic specialisation and divisions of labour, based on economic exchange and professionalisation [137]. Some individuals became healers, just as other individuals took on other political and economic roles [137]. In small-scale societies, these services were likely performed as occasional exchanges, with healers still having to produce their own food [137]. With larger scale societies, individuals may have become increasingly specialised and been able to support themselves through these activities, becoming a ‘profession’ [137].

## COMMUNITY HEALTH BEHAVIOURS

Community health behaviours are the evolutionary roots of human public health practices and institutions. While care behaviours are generally direct interactions between a carer and a recipient, community health behaviours are indirect interactions in which individuals reduce the risk to the group. This can be done through interactions with the environment or through engaging in patterned social interactions (i.e. division of labour, synchronizing hygiene behaviours). That both care and community health behaviours are widespread across the animal kingdom suggests that the two types of behaviours probably have deep, intertwined evolutionary roots.

This evolutionary perspective does not conflict with historical perspectives that credit current public health practices with advances in civic hygiene in the 19th century and modern concepts of disinfection [138–140] and sanitation [141, 142]. While these modern understandings underpinned rapid developments in public health [138–142], they do not undermine the evidence that the precursors to public health already existed in the behavioural repertoire of humans and other animals. Moreover, it is not necessary for a species to have a concept of hygiene in order to benefit from doing it, e.g. nest hygiene in birds [57] and insects [54].

### Environmental protection

Similarly to how self-care evolved before sociality, environmental protection behaviours likely also predate sociality. These are hygiene behaviours in which an individual modifies the physical or biological environment to change the distribution of pathogens in that environment. Importantly, such behaviours are examples of niche construction [39, 143–147]—behaviours (and their consequences) that can mean there is not only genetic inheritance, but inheritance of environmental modifications, the latter of which can have an impact on the selection pressures faced by that species, and by other species living in that environment, such that evolutionary outcomes are different than they would otherwise be (e.g. allow otherwise deleterious traits to persist, or exacerbate and ameliorate competition between species). In this way, niche construction can be considered an independent evolutionary force that has an impact on the evolutionary history of the species living in that environment [146, 147]. In particular, hygienic behaviours can result in the inability of pathogenic species to take hold in the local environment, leading to a reduction in infection rates.

Whereas some healthcare behaviours are direct (in the sense of involving self-care or individual-to-individual interactions), the benefits to others from environmental protection are necessarily ‘indirect,’ as they only involve modifications to the environment in the first instance. The primary motivation of the behaviour is to modify the local niche; it is only the consequences of later interactions with those niche constructions that determine who benefits.

While environmental protection behaviours are widespread, the particular behaviours that are performed can be highly taxon specific, with extreme forms representing convergent evolution. Examples include strategies for reducing pathogens in nests: eusocial insects build antimicrobial/antifungal secretions into the walls of their nests [54], while birds [57, 60] and nest-building mammals [67, 148] may include anti-parasitic materials. Similarly, insects [54] and humans, both of which live at high densities, dispose of their dead (see also reports in mice [149] and wolves [150]). Though these particular behaviours are probably convergent, the proximate mechanisms underpinning them are likely to be multimodal and may vary across taxa according to which senses species use to perceive their environment. For example, insects rely heavily on odour cues to determine when to dispose of the dead (see also mice [149]), while humans likely rely more heavily on behavioural, tactile, and visual cues for recognizing when someone has died. Overall, this pattern suggests that while environmental protection behaviours may be ubiquitous and ancient, some niche dimensions, like nest-building, may exert particularly strong selection for these behaviours, producing the convergences that we see in distant lineages.

In our lineage, the sophistication and scale of niche construction [151] that we engage in—agriculture [152], animal domestication [153, 154], building cities—is a derived state, in that it is far more elaborate than the nests built by nonhuman primates including other apes [155, 156]. Our environmental protection behaviours are also unusually elaborate compared to other species; we build sewer systems, dispose of trash, and purify our water. While cities and other constructed environments did not evolve for the exclusive purpose of pathogen control, constructing them in ways that control pathogens may have contributed to their ability to persist over time. Environmental protection behaviours (Table 1) are fundamental aspects of human public health responses.

### Organizational protection

A final step in the evolution of human care systems came with greater economic specialisation, through organized divisions of labour, institutionalization of care for strangers, and rules for coordinating or synchronising the hygiene behaviours of populations. We call this ‘organisational protection’.

Organisational protection may have some (but likely not all!) of its evolutionary roots in environmental protection. Both types of protection may produce indirect benefits to the broader group and both frequently (but not always!) involve cleaning behaviours. However, the two types of protection have an important difference: organisational protection involves some form of group-level organisation, while environmental protection does not. For example, sanitation behaviours like disinfecting surfaces are environmental protection, however having a specialised subgroup of individuals perform this service for the group (i.e. sanitation workers) is a form of organisational protection. While the boundary between the two can be difficult to define, at their extremes the two concepts are very different. Environmental protection probably originally evolved to benefit the self, even before sociality evolved. Organisational protection requires coordinated patterning of behaviours of individuals in space or time, often via involvement in some institution, which alter the distribution of pathogens, i.e. division of labour or synchronised behaviours of groups. Notably though, **organisational** **protection is not exclusively about the division of labour.** An example of organisational protection which involves synchrony across individuals without a division of labour would be population-wide social distancing. It demonstrates spatial and temporal coordination.

Organizational protection is hypothesised to have been under strong selection during the Neolithic when human populations became more sedentary, engaged in more agriculture [152], and animal domestication [153, 154], and had denser, larger populations [137]. This is argued to have changed the pathogens that humans deal with, potentially increasing the burden of helminths and faecal and water-borne illnesses, and making populations more vulnerable to crowd diseases [157], creating pressure on populations to devise institutions to provide environmental protection services (i.e. rules about cleanliness of water and food, disposal of sewage, etc.).

Stranger care was likely integrated into organisational protection as a type of division of labour. Groups of professional careers, such as nurses and other healthcare workers, started caring for strangers as a full-time activity. There were probably efficiencies involved in embedding both care and protection services in the same institutions since individuals can tend to change from at-risk to sick status without warning. Once instituted, support services such as administration of the institution itself became required as part of delivering protection and care.

A similar form of organisational protection, although not for strangers, is present in some eusocial insect species. In these societies, community health may be undertaken by particular castes who engage in behaviours that provide benefits to the entire colony, such as removing the dead or blocking entry to the colony by diseased individuals [38]. The ways in which organizational protection is delivered differ in important ways in eusocial insects and in humans. In eusocial insects it happens through the behavioural decisions of individuals belonging to the appropriate caste (bottom-up organization), which are stimulated though cues given by the recipient (e.g. chemical [54], behavioural [56], etc.). In humans, organisational protection can emerge through the behaviour of individuals acting on their own initiative (voluntary social distancing by the American public during H1N1 [158]), through community-led mask sharing and protests for border closures during COVID-19 in Hong Kong [159]) and/or through top-down policy directives (i.e. governments [160], see also [161]).

Similarly, the selective pressures driving the evolution of organisational protection in the two taxa likely differ. In eusocial insects, it occurs primarily through kin selection, due to the typically high level of kinship between nest-mates [37]. In humans, it likely occurs through complex and dynamic selective processes, similar to those driving stranger care [136]. This may include multi-level selection in which the individuals benefit, contribute to benefiting their kin, and also benefit unrelated others, creating group-level selection and indirect reciprocity effects [136]. Coordinated behaviours which change the distribution of pathogens in space or time are also likely to be reinforced by network reciprocity [136] and processes of environmental inheritance [143, 144, 146, 147, 151]. Similarly to stranger care, organisational protection also creates significant inter-dependence among participants, and is expected to be fragile to the preconditions for such inter-dependence, such as trust and the reliability of punishment for defectors [162, 163]. This may be why organisational protection, like stranger care, is often professionalised (many public health jobs) and reinforced with forms of immediate benefits like payment. These benefits may be a form of direct reciprocity when the payment comes from the recipient of the service or indirect benefits when it comes from a larger collective (like a town or company). These benefits are immediate in that they are not an emergent benefit, like a reduction in pathogens due to the behaviour of the group.

## INTEGRATION OF CARE AND PROTECTION INTO A ‘HEALTHCARE SYSTEM’

**Figure 3.**
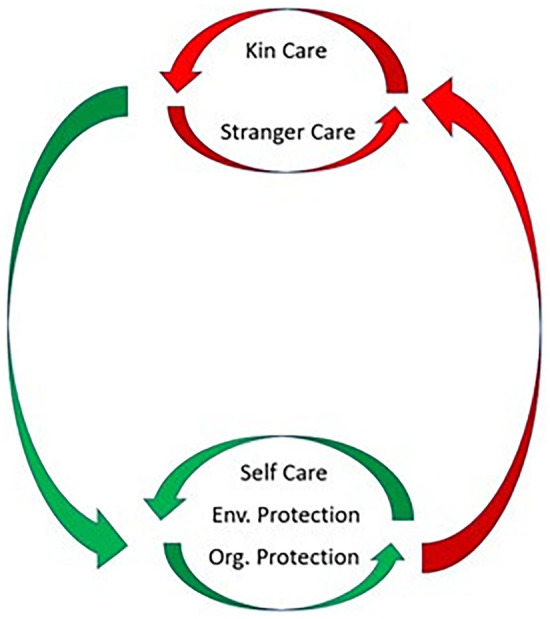
A conceptual diagram showing the hypothesised interactions between the different elements of the human healthcare system. The large circle shows the feedback loops between care mechanisms that decrease exposure risk in the population (green arrow pointing to self-care, environmental protection, organisational protection) and those that may increase it (red arrow pointing to kin care and stranger care). Because kin care and stranger care have the potential to bring susceptible carers in contact with infected individuals, these activities should increase selection for risk reduction (self-care, environmental protection, organisational protection). Feedback loops also exist within these two categories of risk reduction and risk increases. Self-care avoidance, environmental protection, and organisational protection should each decrease the selection pressure for the others, because they all reduce disease spread. Similarly, stranger care should increase the need for kin care, due to increased transmission across kin groups [85]

Understanding how the elements of the healthcare system evolved is important for the field of public health because it enables us to understand when, why, and how the different forms of care and community health behaviours interact to modify disease transmission (Fig. 3). As each element of the healthcare system evolved in interaction with the pre-existing elements (Fig. 2), the older elements of the healthcare system likely supported and facilitated the evolution of the later-evolving elements. This perspective should also help us understand where the system might be fragile or break down (see later discussion).

The addition of each new element to the system has the potential to increase and/or decrease selection on the pre-existing elements. Figure 3 is a conceptual diagram showing the hypothesized interactions between the different types of care and protection. Self-care and environmental protection were early evolving forms of transmission risk reduction (Figs 2 and 3). With the evolution of sociality, individuals engaging in social interactions were more limited in the extent to which they could limit transmission via self-care (i.e. avoidance), but had opportunities to engage in kin care. Kin care produced interactions between susceptible carers and infected individuals, likely increasing selection for risk reduction (self-care, environmental protection, and organizational protection). In humans, trade-offs are also predicted between the two care-networks (kin and stranger). Because stranger care facilitates cross-kin group spread, it is likely to increase the need for kin care [85] (i.e. stranger carers who become infected by the strangers to whom they give care will seek care from kin, as well as possibly other stranger carers). Organisational protection evolved via kin selection in insects and via complex selective pressures in humans (e.g. multi-level selection, indirect reciprocity, etc.). In both taxa, individuals live in large, dense populations which are dependent on one another for food, shelter, extra-maternal care of young [164]). This likely creates intense selection for mechanisms that reduce disease transmission by magnifying the effects of self-care (i.e. avoidance) and environmental protection (i.e. cleaning). Organisational protection encompasses the group-level spatial and temporal patterns through which humans and insects slow disease transmission (i.e. creating a more modular contact structure through divisions of labour or synchronising behavioural defences). In eusocial insects this occurs through the reproductive caste system, in humans frequently via facultative economic specialisation.

Table 1 outlines these similarities in healthcare behaviours; the table is organized by behaviour category. The table shows there are close analogues in behavioural terms between kinds of care and protection in these different animal groups, especially across all groups for the evolutionarily oldest forms like self-care, kin care, and environmental protection. For example, social insects, birds and mammals all perform hygiene behaviours to ensure that their domestic environments are kept clean. It is not surprising that the more recently evolved aspects of the human healthcare system—that is, stranger care and organisational protection—are observed less widely in other species. Organizational protection, which is shared only with eusocial insects, must be a result of convergent evolution, given the great phylogenetic distances between humans and insects. Stranger care appears to be uniquely human. Supplementary File 1 presents a number of hypotheses which can be tested using cross-species studies and/or cross-population studies to support or refute our model for the evolution of the human healthcare system.

## APPLICATION TO COVID-19

Today, humanity is responding to COVID-19 using the human healthcare system. In this section, we first explain how our behavioural responses to COVID-19 are similar to the responses that other species have to infectious diseases (Table 1). Because the different kinds of care evolved under different circumstances, at different points in our evolutionary history, as responses to different kinds of problems, and under different selection pressures, they are unlikely to be perfectly coordinated in their current form. We then show how three types of evolutionary conflicts occur between elements of the healthcare system and inhibit our response to this recent pandemic.

### Applying Figure 3 to our COVID-19 responses

When we examine our responses to COVID-19, it becomes clear that the interactions and feedback loops between the different types of care that are depicted in Fig. 3 also apply to our COVID-19 responses. While many of these observations may seem painfully obvious and trivial in hindsight, this was not the case before the pandemic. As a result, we believe that an explicit discussion of the evolutionary dynamics is worthy of examination—particularly since these processes and feedback loops are still, at the time of writing, ongoing as we experience novel variants and new waves [165, 166].

We would also stress that evolutionary explanations **do not** supersede or negate the importance of political, socioeconomic, historical or cultural differences in populations. Instead, these differences can and should be understood as occurring within the context of this evolutionary framework. The current diversity of healthcare systems exhibited by human populations across the globe are the results of the interactions between selective processes over evolutionary time (Fig. 2) and local processes of niche construction [143], ecological inheritance [143, 144, 151], and cultural evolution [167–169]. This includes both the globally dominant biomedical systems and the systems used by more isolated populations (e.g. [131, 132]). While our study focuses largely on healthcare systems over evolutionary time, this does not dismiss the importance of socio-political or cultural factors—including historical drivers of current structural inequality [170, 171], poverty [172], recent experience with viral epidemics (e.g. [173]). Therefore, the current healthcare system of any group is the result of both its deep evolutionary history and its more recent and rapid political and cultural dynamics (including historical drivers of current structural inequality and poverty, e.g. [170–172, 174]). Although they work on slightly different time scales (biological evolution generally being slower than cultural processes), biology and culture interact and are not entirely independent. In order to fully understand how healthcare systems change and respond to pandemics like COVID-19, we need to understand both the evolutionary and sociocultural processes at work.

In general, feedback loops and trade-offs are visible between the types of care and protection. Countries that rapidly invested in environmental/organisational protection (wearing masks in public, conducting contact tracing and isolating exposed individuals) did not (at least initially) have to resort to the widespread avoidance behaviours seen in other nations (long nation-wide lockdowns), i.e. Taiwan and South Korea [175–177]. Similarly, countries with rapid avoidance strategies (lockdowns & social distancing, i.e. New Zealand and Vietnam [173, 178]) and/or environmental/organisational protection strategies (wearing masks in public, conducting contact tracing and isolating exposed individuals [175–177]) have endured lower care-giving costs and their stranger care facilities (e.g. hospitals and care-homes) were less likely to become overwhelmed like in Italy [179–183]: and New York, USA [184–186], (for review of national comparisons [16, 23]).

Similarly, the feedback loops between kin and stranger care are also visible in our COVID-19 responses. During COVID-19, stranger care facilities were initially major conduits of infection [187], with some nations reporting high numbers of infections occurring in hospitals [188–190] and care homes [191–195] or among healthcare workers [196–198], which can then be seeded back into the community. As stranger care networks become overwhelmed, kin care networks have served a supportive role and absorbed the overflow. At times, people have either been denied stranger care for all but the most severe cases [199–202] or have voluntarily withdrawn due to fears of becoming infected [203–206]. That is, in places where it is known that stranger care is unlikely or a dangerous source of novel infection, sick individuals avoid going to hospitals and experts, and likely rely on kin care. Indeed, we see that once transmission is established within a community, it also travels along kin networks within households [207], with spouses (who likely provide care) and older people being particularly vulnerable [208]. This reliance on kin care instead of expert strangers may be a result of the deeper evolutionary history of kin care.

Many of the behavioural controls employed by societies facing COVID-19 [41] are designed to motivate organisational protection responses in the population. These can include group-level lockdowns [16, 41], changing the contact structure of the population through ‘social bubbling’ [42], shielding vulnerable groups [43], and closing borders [17, 44].

An example of the complex feedback loops currently occurring between these evolutionary/biological and socio-cultural processes is happening as novel COVID-19 variants evolve. Political and cultural differences in how populations contain the virus may make the probability of new variants arising non-random [209, 210]. Populations with higher transmission rates may have a higher likelihood of having novel variants evolve [210]. This exerts biological selection (increased death and disease when variants are more deadly or more transmissible) on that population and also on other populations which may be vulnerable (possibly due to structural inequality in access to healthcare resources including vaccines). Simultaneously, the pandemic is currently exerting pressures for cultural evolution of our healthcare systems, i.e. how do we want to change our healthcare systems to be more robust to the pandemic? Will this involve greater environmental or organisational protection? Increased investment in stranger care facilities like hospitals and vaccine distribution sites around the globe?

The changes that populations make to their healthcare systems are emergent effects of changes in the patterning of healthcare behaviours performed by the individuals in those populations. These emergent effects can produce changes in the separate components (e.g. stranger care) and, through the interactions of the components, on the system as whole (as in Fig. 3). Thus healthcare systems can change as a result of selective processes occurring primarily on individuals, without the healthcare systems themselves needing to reproduce or die like biological entities.

### Conflicts and mismatches within the healthcare system

Despite the long evolutionary history of our healthcare system, it does not operate as a unified whole. The feedback loops described above illustrate the complex web of selective processes operating within the different, interconnected components of the contemporary healthcare system. Here, we describe three types of related conflicts occurring within the system: (1) conflicts based on opposing selection pressures being exerted on individuals, (2) evolutionary mismatches (when the evolved response to a problem no longer matches with the current modern context [211, 212]) and conflicts based on ‘displacements’—when an evolutionarily older form of care is currently dispensed through a more derived form of care, causing the two forms of care to work against each other. While evolutionary mismatches typically occur when the response no longer fits the current modern context [211, 212], here we show that similar conflicts occur when an ancestral response is superseded by a derived response.

**Figure 4.**
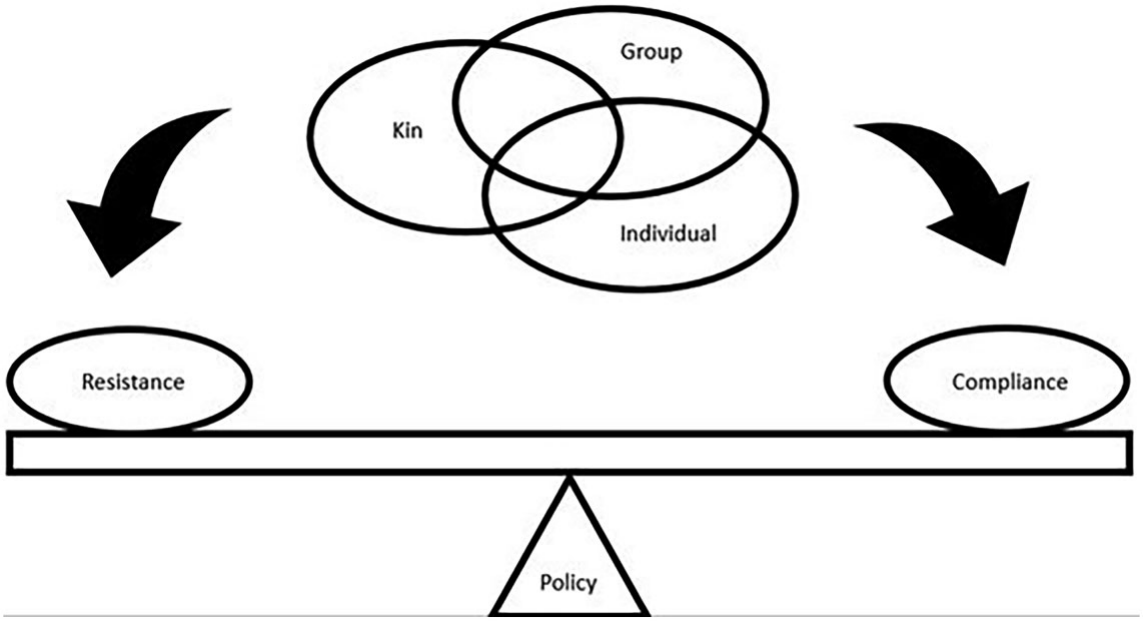
A conceptual diagram showing the multi-level selection pressures that influence whether individuals chose to comply with group-level disease control policies. Compliance or resistance will likely be determined by how well the group-level pressures align or conflict with individual-level and kin-level pressures. Note that the relative importance of the different levels may differ according to life stage, e.g. young adults, parents raising offspring, older people

1. Conflicting pressures at the level of the individual: Figure 4 illustrates the potentially competing selection pressures exerted on individuals as they decide whether to obey or resist government restrictions designed to reduce disease transmission. Individuals balance selective pressures to maximize their individual reproductive fitness (secure resources for survival and reproduction, find mates), maximise their inclusive fitness (keep offspring and kin healthy), and receive benefits from group-level dynamics (contribute to reducing community transmission) [213].
  We can look at these conflicts from the perspective of life-history theory [214, 215], which predicts that individuals should respond to these different selective pressures differently according to their stage of life. This theory can explain some of the dynamics that we see occurring currently [213]. For example, demographic groups that have been criticised for not abiding by social distancing guidelines include young adults [216–220], who are less at risk from COVID-19, may not be caring for offspring, and are under strong selective pressure to prioritise their individual fitness [213]. Similarly, adult workers have resisted staying at home or closing businesses when they have not been accompanied by sufficient financial support to enable businesses to stay afloat and enable them to support their families [221, 222]. In both cases, government policies have requested that individuals prioritise group-level demands over the strong selection pressures to maximise individual and inclusive (kin-based) fitness. Given these strong selective pressures, perhaps the surprising result is not that people violated lockdown orders, but that people abided by them at all.
  It is also worth noting that the balance of these selective pressures will be continually re-evaluated and change over time. In particular, continuing to abide by lockdown orders becomes more costly over time (more missed opportunities for interaction, greater financial losses, while disease risks decrease), causing adherence to decrease. This is not ‘behavioural fatigue [223],’ but a reflection of the changing balance of selective pressures over time.
2. Evolutionary mismatch: Globalisation has produced numerous mismatches between our healthcare behaviours—which evolved in small, local, kin-based communities—and our current globalised environment. We live in a world where people, pathogens, and healthcare resources can move across the world in hours. Yet, repeated waves of COVID-19 have demonstrated that even as newly evolved variants spread globally, we have struggled to build and sustain an effective global response. This includes nations responding slowly to scientific evidence of threats that are far away across the globe, and once the threat is recognised, a failure to distribute resources like vaccines on a global scale (i.e. vaccine nationalism, wealthy nations hording vaccines [224–227]). While the causes of these failures involve complex socio-political dynamics, including histories of structural violence, colonialism, and entrenched inequality [224], they likely also reflect that our healthcare behaviours evolved in small, kin-based communities [85]. As a result, we may have an evolved tendency to focus on local threats and to prioritise giving care to the communities we perceive as our in-group—to the extent that we may ignore more serious, but geographically distant threats like a novel COVID-19 variant or refuse to equitably share resources like vaccines with members of distant communities [224–227]. This understanding of how our evolutionary history may make global coordination fragile, in no way serves to justify our failures [228]. Instead, it highlights the need for actively counteracting these tendencies with information campaigns which emphasise the interconnectedness of our populations today.
3. Evolutionary displacements: This occurs when the typical services of older components of the healthcare system (self-care and kin care) are being dispensed through newer components (stranger care, organisational protection). In humans it reflects an overall trend towards increasingly large-scale, centrally-controlled societies in our species [211, 229]. Table 2 describes a number of displacements that are occurring during COVID-19, causing the different components of the healthcare system to work against each other. An example of a displacement is a shift from primarily personalised, trust-based kin care to impersonal, globalised stranger care.

**Table 2.** Displacements that generate conflicts between different components of our healthcare system during our response to COVID-19

Conflict	Evolutionary context	Current context	COVID conflict examples
Germ theory and biomedical diagnostics	Self-care, kin care	Stranger care, Org. protection	Germ theory [230], biomedical diagnostics, and contact tracing detect cases before people have symptoms that are detectable through face-to-face observations.
Tracking app, contact tracing teams	Self-care, kin care, stranger care	Org. protection	The management of information about who was infected was previously done informally through face-to-face care-giving contacts. It is now done on large scales through specialised groups (contact tracers and apps), sometimes leading to people refusing to be tracked [231, 232].
Care homes	Kin care	Stranger care	Elder care has been outsourced from kin care to stranger care, leading to families demanding to have loved ones released from care homes affected by COVID-19 [233].
Quarantine centres for mild cases	Self-care, kin care	Stranger care	China introduced quarantine centres for mild/moderate cases [234], thus outsourcing this care from self-care & kin care to stranger care, to increase compliance with isolation requirements and reduce intra-family and community transmission.
Psychological trauma in stranger care workers and their families	Stranger care	Org. protection	Stranger care is increasingly dispensed on a greater scale and through dedicated institutions (i.e. hospitals), preventing care-givers from refusing to give care when the risks are too high for themselves and their kin [235]. This can lead to (1) care-givers experiencing trauma [236, 237] or (2) strikes/threats of quitting by carers [238–240].

Human care-giving behaviours likely evolved in small, kin-based communities [85], where care was given within an existing kin relationship or after personally negotiating treatment from a trusted stranger carer (i.e. healer). In the context in which care evolved, trust was likely to be a reasonable proxy for local expertise—kin carers would be in a position to be knowledgeable about the health of their family members and they (and local stranger carers) would potentially also have knowledge about local pathogens and treatments. This is a stark contrast to how our current, globalised healthcare systems operate. Today, medical expertise is rarely a locally-acquired form of knowledge. Biomedical expertise is acquired through years of study and practice in centralised institutions (medical schools, hospitals, etc). As a result, biomedical experts (e.g. doctors) typically have professional, rather than intimate, relationships with their patients. Moreover, a substantial portion of global healthcare, particularly pandemic care, is determined based on the impersonal expertise of international experts who devise large-scale policies that are administered to populations. This means that although we have an evolved preference for kin care, it is being displaced by an impersonal and globalised stranger care system. This displacement can lead to patients valuing (possibly inaccurate) health advice they get from kin or other trusted sources over the expert advice of doctors, and causing kin care and current stranger care systems to work against each other. Vaccine hesitancy based on information from family [241] or religious figures [242, 243] are examples of this occurring during the COVID-19 pandemic.

Improving our understanding of the evolutionary conflicts inherent in healthcare systems will help us to predict and prevent our healthcare systems from breaking down under pressure from pandemics. In particular, a reoccurring finding of this study is that the more recently evolved healthcare behaviours like stranger care and organisational protection (in humans) may have evolved and been maintained by more complex selective pressures than just individual level benefits or kin selection. Interestingly, these behaviours may have evolved in low density, kin-based communities and been maintained through delicate and dynamic balances of multi-level selection and forms of reciprocity, i.e. direct, indirect, and network reciprocity [136]. These evolutionary mechanisms are more fragile and break down due to failures of trust or cooperation. This may explain why we do not see these types of healthcare behaviours in more species and why, in our species, they are frequently reinforced through professionalisation and formalised benefits (payments given directly by the recipient or indirectly from a collective).

## CONCLUSION

In this study, we have developed a novel framework for studying healthcare systems. Our evolutionary approach defines the healthcare system as an emergent result of the interactions between individuals, pathogens, and the environment, making it possible to make comparisons between the care and protective behaviours of different species over evolutionary time. By focusing on the different selective pressures driving different types of healthcare behaviours, we see that there has been both continuity and extensive change over time within and across lineages. Self-care and environmental protection are basal and ubiquitous healthcare behaviours, while kin care is characteristic of social species and stranger care is uniquely human. Organisational protection evolved convergently in eusocial insects and humans. In our lineage it is these evolutionarily younger components that evolved through delicate balances of complex selective pressures (multi-level selection, forms of reciprocity), which have proven the most vulnerable to breaking down.

We believe that this evolutionary perspective provides a powerful framework for understanding and predicting how healthcare systems evolve in response to selective pressures—both in the past and how they may continue to change in the future. This is relevant for understanding care-seeking and care-delivery at individual, family, institutional, and group (community/national/international) levels. Ours is one of the few approaches to note that there are various kinds of conflicts of interest operating within and between levels of organization that can impact on the effectiveness of messaging and policy directives intended to influence behaviour at these levels. Moreover, this application has illuminated two additional types of conflicts (evolutionary mismatches and displacements) within healthcare systems which may contribute to the fragilities of, and failures in, pandemic responses. Adequate management of these conflicts is necessary to ensure efficient responses to some healthcare problems, especially in cases of emergencies like pandemics.

We argue that our framework is an important backdrop as populations consider if and how to change their health systems to be more robust to COVID-19 and future pandemics. A major contribution of our study is to highlight the evolutionary origins of some of the conflicts that are occurring (conflicting selective pressures on individuals, evolutionary mismatches between the context in which our healthcare behaviours evolved and our globalized world of today, and displacements between different types of care). We hope that this perspective will enable us to better understand how these evolutionary conflicts interact with, and potentially compound, the socio-political factors that may hinder our pandemic responses (e.g. global inequalities) and to evaluate what future changes to our health systems would be beneficial and possible.

## Supplementary data

Supplementary data is available at *EMPH* online.

**Conflict of interest:** None declared.

## Supplementary Material

eoac004_Supplementary_Data
