# Supplementary material for: The evolution of the human healthcare system and implications for understanding our responses to COVID-19
Source: Evol Med Public Health. 2022 Feb 12;10(1):87–107. doi: 10.1093/emph/eoac004 (PMC8908543; doi:10.1093/emph/eoac004)
Supplement: eoac004_Supplementary_Data [file eoac004_supplementary_data.docx]

# Supplementary Appendix: Testable Hypotheses

## Theory-derived hypotheses testing the evolution of healthcare systems

The postulated evolution of each of the components of the healthcare system, and how they fit together, suggests a number of testable hypotheses, a sample of which follow. These hypotheses use cross-species studies and/or cross-population studies to test our model for the evolution of healthcare systems.

**Caring for the contagious hypothesis:** If care for individuals with noninfectious conditions and injuries supports the evolution of care for infectious conditions, the two should be correlated. Across species, and within species, populations with higher rates of injury/noninfectious conditions should see higher rates of care (as opposed to avoidance) for those with infectious conditions (kin or stranger care). Alternatively, if injuries/noninfectious conditions do *not* support the evolution of care for the infected, we would predict that it would increase selection for discriminating between the infectious/noninfectious conditions. In this case we would expect to see species/populations with high rates of injuries/noninfectious conditions discriminate and provide care only in those circumstances. Note that these alternatives may not be entirely mutually exclusive.

**Evolution of environmental protection hypothesis:** If it is linked with niche construction, then the types of environmental protection should correlate with the degree to which niche construction is engaged in (across species and populations). So species/populations with greater niche construction activities should engage in more environmental protection as part of that activity.

**Evolution of kin care hypothesis:** If kin care evolved together with infant rearing systems, the types of care given to young and to the sick (across species and populations) should be similar. In particular, increases in the degree of offspring care should correlate with increases in the level of care for the sick.

**Evolution of stranger care hypothesis:** Across human populations, when stranger care networks become conduits for infections, we should see increases in stranger care correlate with increases in kin care (to deal with the increased spread of disease to kin).

**Direct care vs risk reduction hypothesis:** Also, across populations and species, greater direct care-giving (kin/stranger) should correlate with combinations of increases in environmental protection, self care (avoidance) or organisational protection (Figure 3). Which of these it is, will likely depend on the degree of sociality. Individuals of highly social species will be unable to isolate themselves effectively enough to prevent disease transmission. Therefore, they are predicted to use environmental and organisational protection because the benefits can occur through emergent effects of behaviours of many individuals. Less social species with smaller contact networks should use self care (avoidance).
